# Vancomycin-Lipopeptide Conjugates with High Antimicrobial Activity on Vancomycin-Resistant Enterococci

**DOI:** 10.3390/ph13060110

**Published:** 2020-05-29

**Authors:** Eric Mühlberg, Florian Umstätter, Cornelius Domhan, Tobias Hertlein, Knut Ohlsen, Andreas Krause, Christian Kleist, Barbro Beijer, Stefan Zimmermann, Uwe Haberkorn, Walter Mier, Philipp Uhl

**Affiliations:** 1 Department of Nuclear Medicine, Heidelberg University Hospital, Im Neuenheimer Feld 400, 69120 Heidelberg, Germany; eric.muehlberg@web.de (E.M.); Florian.Umstaetter@med.uni-heidelberg.de (F.U.); Andreas.Krause@med.uni-heidelberg.de (A.K.); Christian.Kleist@med.uni-heidelberg.de (C.K.); Barbro.Beijer@med.uni-heidelberg.de (B.B.); Uwe.Haberkorn@med.uni-heidelberg.de (U.H.); Walter.Mier@med.uni-heidelberg.de (W.M.); 2 Institute of Pharmacy and Molecular Biotechnology, Heidelberg University, Im Neuenheimer Feld 364, 69120 Heidelberg, Germany; Domhan@uni-heidelberg.de; 3 Institute for Molecular Infection Biology, University of Würzburg, Josef-Schneider-Straße 2/D15, 97080 Würzburg, Germany; tobias.hertlein@uni-wuerzburg.de (T.H.); knut.ohlsen@uni-wuerzburg.de (K.O.); 4 Department of Medical Microbiology and Hygiene, Heidelberg University Hospital, Im Neuenheimer Feld 324, 69120 Heidelberg Germany; Stefan.Zimmermann@med.uni-heidelberg.de; 5 Clinical Cooperation Unit Nuclear Medicine, German Cancer Research Centre (DKFZ), Im Neuenheimer Feld 260, 69120 Heidelberg, Germany

**Keywords:** antibiotics, multidrug-resistant bacteria, enterococci, vancomycin, structural modification, fatty acids, polycationic peptides

## Abstract

Multidrug-resistant bacteria represent one of the most important health care problems worldwide. While there are numerous drugs available for standard therapy, there are only a few compounds capable of serving as a last resort for severe infections. Therefore, approaches to control multidrug-resistant bacteria must be implemented. Here, a strategy of reactivating the established glycopeptide antibiotic vancomycin by structural modification with polycationic peptides and subsequent fatty acid conjugation to overcome the resistance of multidrug-resistant bacteria was followed. This study especially focuses on the structure–activity relationship, depending on the modification site and fatty acid chain length. The synthesized conjugates showed high antimicrobial potential on vancomycin-resistant enterococci. We were able to demonstrate that the antimicrobial activity of the vancomycin-lipopeptide conjugates depends on the chain length of the attached fatty acid. All conjugates showed good cytocompatibility in vitro and in vivo. Radiolabeling enabled the in vivo determination of pharmacokinetics in Wistar rats by molecular imaging and biodistribution studies. An improved biodistribution profile in comparison to unmodified vancomycin was observed. While vancomycin is rapidly excreted by the kidneys, the most potent conjugate shows a hepatobiliary excretion profile. In conclusion, these results demonstrate the potential of the structural modification of already established antibiotics to provide highly active compounds for tackling multidrug-resistant bacteria.

## 1. Introduction

An increasing number of antibiotic-resistant bacterial strains are frequently being reported all over the world [1]. The spread of multidrug-resistant bacteria, such as methicillin-resistant *Staphylococcus aureus* (MRSA) and vancomycin-resistant enterococci (VRE), is threatening, and has forced the World Health Organization (WHO) to categorize them as “high priority” pathogens [2]. Recent studies have estimated that the annual deaths caused by multidrug-resistant bacteria in the EU and the European Economic Area in 2015 were up to 33,000, thereby exceeding the collective number of deaths caused by influenza, tuberculosis, and HIV [3]. Further reports have estimated that there are 700,000 deaths worldwide every year due to bacterial infections [4]. Since the beginning of the 20th century, the mortality rate of bacterial infectious diseases in the United States of America has decreased significantly [5]. This trend can be associated with the discovery of the β-lactam antibiotic penicillin in 1928 and more than 20 other classes of antibiotics thereafter, which have been designed and approved [5,6,7,8].

Glycopeptides still represent one of the most important classes of antibiotics, and are also often considered as a last resort therapeutic option for multidrug-resistant Gram-positive bacteria. The first representative of this class, vancomycin, was discovered in the fermentation broth of the Gram-positive filamentous actinomycete *Amycolatopsis orientalis* in 1952 by E.C. Kornfield [9,10]. From then on, it was used for the treatment of infections with Gram-positive bacteria, particularly severe infections with methicillin-resistant *Staphylococcus aureus* [11]. Vancomycin exerts its antimicrobial activity by targeting the cell wall synthesis of replicating Gram-positive bacteria [12]. By forming a complex with the D-Ala-D-Ala C-terminus of peptidoglycan precursors, which are the basic building units of the cell wall, vancomycin prevents its further processing by the enzyme transglycosylase [13]. Therefore, the assembly of a functional cell wall is inhibited, making the exposed bacteria vulnerable to external influences such as osmotic pressure [14]. For over 30 years, vancomycin was a reliable last resort treatment option [15]. However, since 1988, when the first vancomycin-resistant enterococci (VRE) (*Enterococcus faecium* and *Enterococcus faecalis*) appeared, an increasing number of nosocomial enterococci isolates have been reported to exhibit vancomycin-resistance [16]. These VRE also include multidrug-resistant strains which pose a great threat to exposed humans, especially for patients in intensive care units and persons with a suppressed immune system [17]. The resistance against vancomycin is achieved by modification of the peptidic binding motif. Depending on the type of resistance, these bacteria develop either a D-Ala-D-Lac (*vanA*, *vanB*) or D-Ala-D-Ser (*vanC*) terminus, which results in a massive loss of vancomycin binding [18]. Once bacteria show resistance to the standard set of antibiotics, further potent substances are urgently required [19]. Unfortunately, in most cases, therapeutic options are limited [20]. It is highly important to know that VRE infections usually manifest as surgical site or organ/space infections, including biliary tract infections and intra-abdominal abscesses in liver transplant recipients [21]. Currently, no bactericidal antibiotic is available for the effective treatment of these infections, as tigecycline is only bacteriostatic, while daptomycin exhibits a poor pharmacokinetics profile to liver and bile.

The growing need for new antibiotic treatments against multidrug-resistant bacteria is currently not covered by commercially available antibiotics [22]. New substances must be developed to compete with steadily evolving multidrug-resistant bacteria. Different approaches for combatting bacterial infections have recently been published. These approaches cover monoclonal antibodies, antimicrobial peptides, quorum-sensing inhibitors, bacteriophages, and metallic or polymeric nanoparticles [23]. Other approaches focus on the structural modifications of known antibiotics, such as vancomycin, to reactivate their potency or alter their in vivo characteristics. Promising modification strategies represent, for example, cationic sulfonium moieties and single cationic quaternary ammonium charges combined with saturated fatty acids, as well as chlorobiphenyl and dipicolyl extensions [23,24,25,26,27,28].

**Figure 1:**
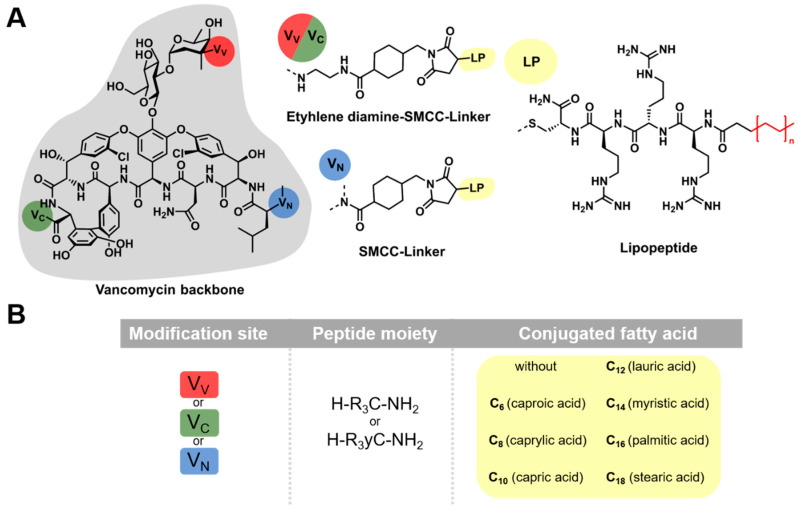
Schematic illustration of vancomycin-lipopeptide conjugates. (**A**) Three different modification sites of vancomycin were addressed. The primary amine position V_V_ (red) and the carboxylic acid position V_C_ (green) were extended by a combination of ethylene diamine and the heterobifunctional crosslinker succinimidyl 4-(*N*-maleimidomethyl)cyclohexane-1-carboxylate (SMCC). The secondary amine position V_N_ (blue) was extended with SMCC only. The maleimide function of SMCC enables the coupling of cysteine-containing lipopeptides (LP, yellow) via thiol maleimide Michael addition. For this coupling, peptide sequences containing cysteine (C) and tri-arginine (R_3_), combined with saturated fatty acids of varying chain lengths, were used. (**B**) Nomenclature of the generated conjugates. Conjugates are always designated in the described order: modification site (V_V_, V_C_, and V_N_)—peptide moiety (one-letter notation)—conjugated fatty acid.

Further approaches have focused on the structural modification of vancomycin with poly-arginine/poly-cationic peptides. A D-octaarginine conjugate of vancomycin eliminated biofilm-associated MRSA in a murine wound infection model, MRSA persister cells, and vancomycin-resistant enterococci [29]. Efficacy against Gram-negative bacteria was achieved by single amino acid conjugation [30] and a therapeutic effect in a systemic infection mouse model was also demonstrated [31]. Another recent approach has dealt with the conjugation of fatty acids to the basic core of vancomycin by small peptide linkers. The peptide linkers used to obtain the most effective conjugates mainly consisted of the amino acid lysine. These cationic effector sequences, in combination with lipophilic moieties, are supposed to increase the affinity of the conjugates to the overall negatively charged bacterial cell membrane [32]. The already approved lipoglycopeptides dalbavancin and telavancin differ in terms of the positioning and in the way of coupling the lipophilic (fatty acid) part, since these substances bear no peptide sequence between the vancomycin core and the lipophilic component. Additionally, resistance development has already been described for these drugs, making further research indispensable [33]. The impact of fatty acids on vancomycin conjugates has also been addressed in recent publications, demonstrating the relevance of this modification strategy [26,34]. Furthermore, an evaluation of the antimicrobial activity of arginine-rich lipopeptides and homo-arginine peptides against Gram-positive bacteria showed promising results [35,36]. Based on these previous findings, we investigated the structure–activity relationship of vancomycin lipopeptide conjugates, depending on the modification site and fatty acid chain length. We combined fatty acids of varying chain lengths (between six (caproic acid) and eighteen (stearic acid) carbon atoms) with polycationic peptides (mainly tri-arginine peptide moieties) (Figure 1). The coupling strategy and nomenclature (V_N_, V_V_, and V_C_) were applied as previously described by Umstätter et al. [31].

To examine the antimicrobial potential of these conjugates with respect to their modification site and increasing lipophilicity, the antimicrobial activity of the lipopeptides was evaluated by determination of the minimum inhibitory concentration (MIC; broth microdilution assay) [37,38,39]. The most active conjugate (V_V_-R_3_C-C_12_) during MIC testing was further characterized in vivo by scintigraphic imaging and biodistribution studies.

## 2. Results

**Figure 2:**
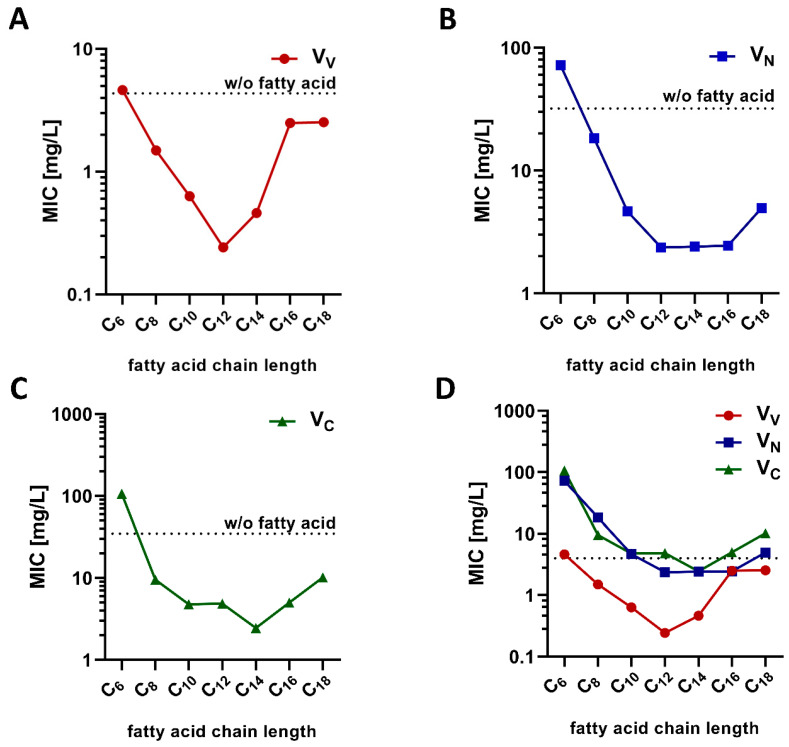
(**A**–**C**) Antimicrobial activity of vancomycin-lipopeptide conjugates (different modification sites) for a clinical isolate of *Enterococcus faecium* (UL602570). The dotted line indicates the minimum inhibitory concentration (MIC) of the respective conjugate modified only with the peptide sequence. (**D**) Comparison of all three modification sites. The dotted line represents the enterococci resistance breakpoint. Data are shown as the median of a minimum of three independent experiments tested at least in duplicate. A tabular overview is shown in the supplementary information, Table S1.

All conjugates used to determine the influence of varying fatty acid chain lengths could be readily synthesized in sufficient amounts for subsequent experiments in this study. After synthesis and purification, the MIC of each conjugate was determined on a resistant clinical isolate of *E. faecium* (UL602570; *vanA*; resistance against vancomycin up to 640 mg/L). The results obtained (Figure 2) demonstrated a high variability in the antimicrobial potential (as shown by the varying MIC values), depending on the fatty acid chain length. Interestingly, the MIC values decreased upon shortening the fatty acid chain length till C_12_ (lauric acid). Therefore, the most potent conjugates consisted of twelve or fourteen carbon atoms (fatty acid chain length). Further shortening of the lipophilic moieties decreased the antimicrobial potential, as demonstrated by the increasing MIC values. With respect to lauric acid, V_V_ and V_N_ modifications showed the lowest MIC values, also demonstrating the influence of the modification position. In general, conjugates modified at the V_V_ position showed the lowest MIC, and thus the highest antimicrobial potential against *E. faecium*. Therefore, this modification site and conjugates containing lauric acid were chosen for subsequent characterization experiments.

**Figure 3:**
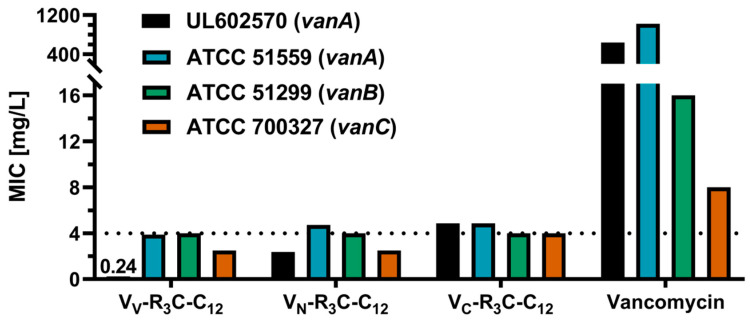
Comparison of MIC values for vanA, vanB, and vanC resistant enterococci. The tested conjugates were modified with the same lipopeptide, but coupled on varying modification sites. The different MIC values demonstrate the influence of the modification position. The dotted line denotes the enterococci resistance breakpoint of 4 mg/L. Data are shown as the median of a minimum of three independent experiments tested at least in duplicate. A tabular overview is shown in the supplementary information, Table S2.

The V_V_-modified conjugate showed the lowest MIC values, although the difference between the vanA-type of resistant *E. faecium* strains was higher compared to the other conjugates (Figure 3). Nevertheless, all modifications showed an increased antimicrobial potential when compared to unmodified vancomycin, as demonstrated by the highly decreased MIC values.

**Figure 4:**
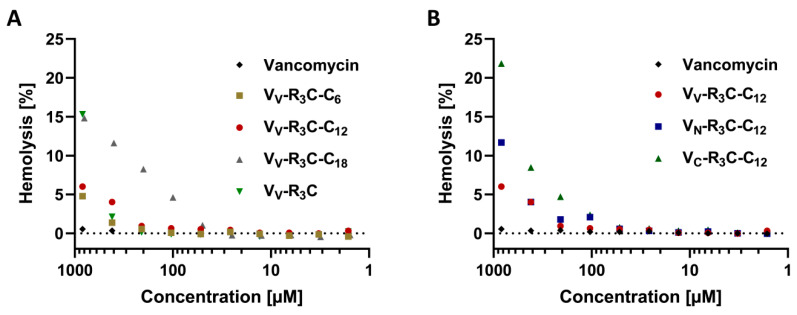
Hemolysis study of selected conjugates in comparison to vancomycin. The hemolytic activity of selected conjugates was investigated via a hemoglobin release assay. The concentrations applied ranged between 1 and 900 µM. (**A**) V_V_-modified conjugates are compared with vancomycin. (**B**) The same modification at the three different modification sites is compared with vancomycin. All tested conjugates showed no relevant hemolytic effect in target concentrations (below 2 µM). Data (% lysis relative to complete lysis by TRITON^®^X-100) are shown as the mean of three independent experiments tested in duplicate.

Based on these promising results and to enable subsequent in vivo experiments, the most active conjugates were further characterized with respect to potential cytotoxic effects. In order to investigate the hemolytic behavior, human blood from healthy volunteers was taken and exposed to different concentrations of selected conjugates. As a control, unmodified vancomycin, known to have non-hemolytic properties up to 12 mM [40], was used (Figure 4).

All tested conjugates showed no hemolytic activity at tested concentrations of up to 100 µM (Table S3), which is up to 50-fold higher than the MIC (all conjugates showed MIC values below 2 µM) and many times higher than a hypothetical dosage (this estimation was based on a typical vancomycin dosage and the determined MIC values) [33]. Based on this knowledge, the slight hemolytic effect observed at higher concentrations could be neglected. Nevertheless, this described hemolytic effect could neither be observed for the original substance vancomycin nor for the previously published polycationic peptide conjugates [31].

It is known from previous publications that polyarginine-modified vancomycin conjugates are mainly excreted through the liver [31]. To exclude cytotoxic effects of the novel conjugates on this organ site, they were tested on human liver cells. Here, concentrations around eight-fold higher than the MIC of the respective conjugate were used. At these concentrations, none of the tested conjugates displayed cytotoxic effects (Table S4). Therefore, these conjugates were considered suitable for in vivo studies. In these in vivo studies, no signs of acute cytotoxic effects were observed, enabling imaging studies up to at least 96 h post injection. At the termination of these studies, again, no signs of acute cytotoxic effects in animal behavior could be observed.

Subsequently, the antimicrobial potential of the most active conjugates was examined on the resistant strain *E. faecium* (ATCC 51559, *vanA*, being resistant to up to 1024 mg/L). Additionally, the antimicrobial potential on vanB- and vanC-resistant strains, namely *E. faecalis* ATCC 51299 and *Enterococcus casseliflavus* ATCC 700327, was assessed, as they also show resistance against glycopeptide antibiotics (Figure 3). The higher resistance of *E. faecium* ATCC 51559 to vancomycin is transferable to most of the tested conjugates. While both the V_N_- and V_V_-modified conjugates showed a lower antimicrobial potential against *E. faecium* ATCC 51559, the V_C_-modified conjugate did not show this differentiation. All three conjugates displayed the same activity against the resistant enterococci (*vanB*; MIC values being in proximity to the resistance breakpoint). The V_V_- and V_N_-modified conjugates were slightly more effective against resistant *E. casseliflavus* (*vanC*) compared to the V_C_-modified conjugate.

**Figure 5:**
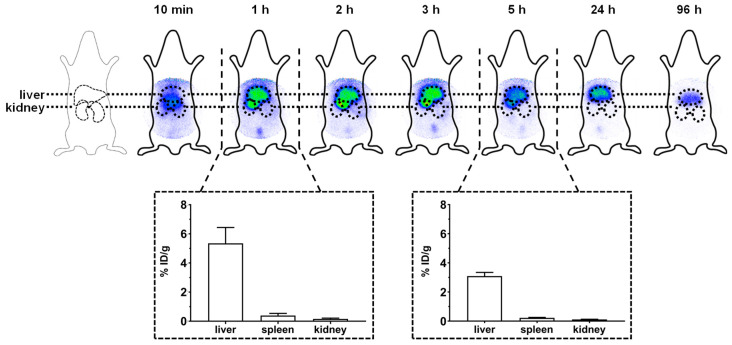
Scintigraphic imaging and biodistribution studies of V_V_-R_3_yC-C_12_. After an intravenous injection of the labeled conjugate, images were taken at several time points (10 min, 1 h, 2 h, 3 h, 5 h, 24 h, and 96 h) post injection. In an additional experiment, further Wistar rats were injected intravenously with the radiolabeled compound and euthanized after 1 and 5 h. Subsequently, the organs were removed to determine the percentage of the injected dosage per gram organ (% ID/g). Data is shown within the dotted squares (n = 3).

The pharmacokinetics of the most potent conjugate V_V_-R_3_C-C_12_ was investigated by radiolabeling and subsequent molecular imaging in female Wistar rats. For radiolabeling purposes, the peptide sequence was extended by an additional D-tyrosine, as described previously [41]. The results of the molecular imaging and biodistribution studies are shown in Figure 5.

The scintigraphic images show the expected hepatobiliary excretion profile of the V_V_-R_3_yC-C_12_-conjugate. After 10 min, circulation in the blood is still visible, while all subsequent images show primarily enrichment within the liver. To further determine the excretion of the conjugate from the liver, scintigraphic imaging was also performed 1 and 4 days post administration. The respective images show the clearance of the main part of the conjugate by the liver, as demonstrated by the decreasing intensity of the radioactive signal.

## 3. Discussion

When compared to vancomycin, the synthesized vancomycin-lipopeptide conjugates showed increased antimicrobial activity on vancomycin-resistant enterococci, depending on the fatty acid chain length. The data obtained indicate that modification with lauric acid (C_12_) enables the highest efficacy on vancomycin-resistant enterococci. These findings are concordant with previously published studies, which also described lauric acid as the most inhibitory fatty acid against Gram-positive bacteria or as the best compromise between antimicrobial activity and cytotoxicity [35,42]. Modifications with longer fatty acids tend to decrease the MIC of the conjugates. Besides, these longer lipophilic moieties also impede the synthesis of the conjugate due to their poor solubility in aqueous solutions. This poor solubility might further limit the use of these conjugates for in vivo experiments.

In contrast to the recently published study of Umstätter et al. [31], describing the secondary amine (V_N_) modification site as the method of choice for modification with polycationic peptides, in this study, the lipopeptide modifications at the primary amine (V_V_) site seem to be more effective compared to V_N_ and V_C_ modification. Subsequent experiments showed the resistance breaking potential of these conjugates against the three most common types of vancomycin-resistance (*vanA*, *vanB*, and *vanC*). For the structurally related lipoglycopeptide antibiotics dalbavancin and telavancin, not carrying the described cationic effector sequence [32], no resistance breakpoint is specified for vancomycin-resistant enterococci, as there is insufficient evidence for their efficacy [33]. Studies on vancomycin-resistant and -susceptible strains with these two antibiotics resulted in a broad range of antimicrobial activity [43]. Interestingly, the novel lipopeptide conjugates seem to be less effective on the low-level resistant *E. casseliflavus* than the previously reported polycationic peptides [31]. However, it has to be considered that this low-level resistance (*vanC*) is associated with an altered cell wall precursor (D-Ala-D-Ser) in comparison to the other types of resistance [18]. Therefore, the aim of future research should be to test a broader spectrum of different vancomycin-resistant bacteria with a special focus on low-level resistances.

Although the exact mechanism of action cannot currently be provided, a hypothesis can be generated based on approaches already published [29,31,32]. For the previously published lipoglycopeptides, the vancapticins, the mechanism of action is assumed to be an interplay of the cationic effector sequence, targeting the negatively loaded bacterial cell wall and the lipophilic tail anchoring the conjugate in the bacterial cell wall [32]. Furthermore, the cationic peptide might enhance the delivery of the compound through the barrier [29,44]. Based on hemolysis studies, a simple lysis of cells can be excluded, as previously stated for other peptide-vancomycin conjugates [31].

The synthesized conjugates displayed no relevant hemolytic effect at concentrations much higher than the determined MIC values and at hypothetical clinical dosages. Even if the hemolytic effect is negligible, it seems conceivable that the fatty acid might disrupt the membrane of the red blood cells. In addition, the influence of the coupling position on hemolytic activity should be investigated as the derivative V_V_-R_3_C showed slight hemolytic effects at higher concentrations. The conjugates also showed no cytotoxic effect on liver cells at concentrations up to eight times higher than the MIC. The in vivo experiments in female Wistar rats also demonstrated the good cytocompatibility, as no remarkable change in animal behavior could be observed up to 96 h after an intravenous injection of the conjugate. In these studies, a noticeable change in the biodistribution profile could be observed. As expected, the most promising conjugate (V_V_-R_3_yC-C_12_) tested in biodistribution studies revealed a mainly hepatobiliary excretion profile, as previously described for polycationic peptide conjugates [31]. Currently, a treatment gap for tackling VRE infections such as biliary tract infections or liver abscesses, especially in liver transplant recipients, exists [21]. The conjugates described in this study, with their excellent antimicrobial effect against VRE and favorable pharmacokinetics to the liver and bile, might serve as potential candidates to cover this therapeutic need. This applies, in particular, to V_V_-R_3_C-C_12_, with its outstandingly long liver enrichment. In general, this study demonstrated a strong impact of the fatty acid chain length and the coupling position on the antimicrobial activity of the conjugates. Based on these findings, an influence of the peptide sequence can also be assumed. Therefore, for subsequent studies, the effect of an amino acid exchange (e.g., exchange of arginine by lysine) should be considered.

## 4. Materials and Methods

All Fmoc-L-amino acids were purchased from Orpegen Peptide Chemicals GmbH, Heidelberg, Germany. Fmoc-D-tyrosine, as well as the rink amide resin, were obtained from Iris Biotech GmbH, Marktredwitz, Germany. Caproic acid, capric acid, and palmitic acid were obtained from ICN Biomedials Inc, Irvine, CA, USA. All other fatty acids were purchased from Sigma-Aldrich Chemie GmbH, München, Germany. Vancomycin.HCl was obtained from Noridem Enterprises Limited, Nicosia, Cyprus, or Hikma Pharma GmbH, Planegg, Germany. Ethylene diamine for the site-specific derivatization of vancomycin was purchased from Lancaster, Mühlheim am Main, Germany. Sulfo-SMCC (4-(*N*-Maleimidomethyl)cyclohexane-1-carboxylic acid 3-sulfo-*N*-hydroxysuccinimide ester) for crosslinking was obtained from Carbosynth, Limited, Compton, Berkshire, UK or Iris Biotech GmbH, Marktredwitz, Germany. Purification of the products was performed by preparative HPLC using a LaPrep P 110 (VWR International, Karlsruhe, Germany) HPLC system equipped with a Reprosil™ Gold 120 C-18 column (4 μm, 150 × 20 mm; Dr. Maisch HPLC GmbH, Ammerbuch, Germany). Analyses were performed by LC/MS using a Thermo Scientific Exactive mass spectrometer. Vancomycin.HCl (potency 99.8%) used as a control in antimicrobial testing was purchased from Sigma-Aldrich Chemie GmbH, München. The cell number in microdilution assays was adjusted using a McFarland-counter DensiCHEK^®^ plus, from bioMerieux, Marcy-l′Étoile, France. The antimicrobial activity was determined in microdilution assays using polypropylene, in U-bottom 96-well plates obtained from Greiner Bio-One GmbH, Frickenhausen, Germany. Mueller-Hinton-Broth II (cation-adjusted) was obtained from Sigma-Aldrich Chemie GmbH, Steinheim, Germany. All described clinical isolates were obtained from the Institute for Medical Microbiology and Hygiene, Heidelberg University Hospital, Heidelberg, Germany. All other reference strains were supplied by the Department of Infectious Diseases, Medical Microbiology and Hygiene, Heidelberg University, Heidelberg, Germany. For absorbance measurements in hemolysis, an Infinite M200 PRO microplate reader, from Tecan Trading, Maennedorf, Switzerland, was used. V-bottom and flat-bottom polystyrene plates used in hemolysis assays were bought from Greiner Bio-One GmbH, Frickenhausen, Germany. The control in hemolysis assays TRITON^®^ X-100 was purchased from Sigma Aldrich, Steinheim, Germany. ^125^I was obtained from Hartmann Analytic GmbH, Braunschweig, Germany. Rats for biodistribution and molecular imaging were purchased from Janvier labs, Le Genest-Saint Isle, France. For scintigraphic images, a γ-camera (Gamma Imager, Biospace Lab, Paris, France) was used and for biodistribution studies, the remaining radioactivity in the specific organs was measured with a Cobra Auto γ-Counter, from Packard BioScience Co., Meriden, CT, USA.

### 4.1. Experimental Section

All analytical data were obtained by reverse-phase high performance liquid chromatography (RP-HPLC) on a C18 column (Chromolith^®^ Performance RP-18e, 100 × 3 mm) coupled to an Agilent 1100 series system with UV detection at 214 nm and subsequent LC-MS using a RP-HPLC Chromolith^®^ Performance RP-C18e column (100 × 3 mm) coupled to an Agilent 1200 series system. Mass detection (MS) was performed on a Thermo Fisher Exactive Orbitrap MS system.

All purification steps were performed by semi preparative RP-HPLC. A Reprosil Pur 120 C18-AQ, 5 μm (250 × 25 mm) was coupled to a Gilson 331 series system with UV detection at 214 nm. Substances were dissolved and subsequently separated on a linear gradient of H_2_O (0.1% TFA) and MeCN (0.1% TFA) for 15 or 25 min, respectively. The gradient was adjusted for every substance to fit the characteristics. Afterwards, the purified substances were lyophilized for 16–24 h using an Alpha 2-4 LD plus system.

#### 4.1.1. Synthesis of the (Lipo-)Peptides

Peptide synthesis was performed as described previously using a standard protocol with the Fmoc-strategy and HBTU activation [45]. Briefly, the Fmoc-Rink-Amide resin (loading of 0.67 mmol/g) was loaded with Fmoc-cysteine to obtain the carboxyl-terminal cysteine residue. The following amino acids were each coupled for 1 h. After coupling had been completed, the resin was washed and dried in vacuum.

For coupling of the fatty acids, 8 eq. of the respective fatty acid, as well as 7.8 eq. HBTU, were dissolved in DMF. Then, 24 eq. *N*-ethyl-*N*-(propan-2-yl)propan-2-amine (DIPEA) was added. After 5 min, the solution was incubated with the dried resin for 1 h. After coupling had been completed, the resin was washed again and dried in vacuum. The final cleavage of the (lipo-)peptides was performed for 2 h using TFA, TIS, and H_2_O (95/2.5/2.5 *v*/*v*/*v*, 1 mL per 100 mg resin). (Lipo-)peptides were precipitated in diethyl ether and dried in vacuum. Analysis and purification were performed as described above.

#### 4.1.2. Vancomycin-Conjugate Synthesis

To address the different coupling positions of vancomycin, the previously described strategies were applied [31]. Briefly, for the respective derivatives (namely V_V_, V_C_, and V_N_), the following approaches were employed.

The amino function of the vancomycin sugar moiety was addressed using the strategy described by Long et al. [46]. A preliminary substance was synthesized using vancomycin hydrochloride and *N*-(9-fluoroethoxycarbonyl)glycinal and purified by preparative HPLC. This structure was deprotected with quinuclidine and used without further purification. For the synthesis of V_V_-modified vancomycin, this precursor substance was dissolved in PBS (pH 8.16) and mixed with a Sulfo-SMCC in DMSO stock solution. The reaction mixture was purified after 30 min by preparative HPLC.

To conjugate the linker moiety to the V_C_ position, the carboxyl function had to be functionalized by ethylene diamine. Therefore, vancomycin hydrochloride was dissolved in DMSO and 3 eq. of *N*-ethyl-*N*-(propan-2-yl)propan-2-amine (DIPEA) was added. This solution was subsequently mixed with ethylene diamine, benzotriazol-1-ol (HOBt), and (7-azabenzotriazol-1-yloxy)tripyrrolidinophosphonium hexafluorophosphate (PyAOP). After the reaction had finished, the mixture was purified and analyzed as described above and lyophilized for storage reasons. The V_N_ position could be addressed without further modification.

For linker coupling, the respective vancomycin derivative (V_V_, V_C_, and V_N_) was dissolved in PBS (pH 8.16). A total of 0.5 eq. of the bifunctional linker Sulfo-SMCC was dissolved in DMSO and added. The mixture was shaken at room temperature for about three hours. After the reaction had finished, the mixture was lyophilized for storage reasons. For purification, the dried product was resolved in water and purified and analyzed as described before.

Coupling of the (lipo-)peptide residue was performed following the previously described strategy [31]. To enable Michael addition, 1 eq. of vancomycin-linker derivative was dissolved in PBS (pH 5.5) and mixed with 2 eq. of the peptide or lipopeptide, dissolved in PBS (pH 5.5). After 2 h, the substances were immediately purified by semi preparative RP-HPLC, as described previously.

#### 4.1.3. Antimicrobial Activity Testing

The minimum inhibitory concentration (MIC) was determined by microdilution following the EUCAST and CLSI guidelines via the broth microdilution assay on a 96-well plate [37,38,39]. Substances were dissolved in saline solution (0.9%) at a concentration equimolar to 1.28 mg/mL vancomycin. With an increasing hydrophobicity, some substances had to be pre-dissolved in DMSO. The final concentration of DMSO remained below 1.5% and was compared to the DMSO control (no antimicrobial effect was observed up to the highest concentration tested (10%)). All substances were serially diluted on the 96-well plate using Mueller-Hinton-Broth II (cation adjusted). Vancomycin was always used as a control. For bacterial loading, an overnight culture of the referring strain was taken and adjusted to a turbidity corresponding to 10^8^ cfu/mL. The bacterial suspension was further diluted to load a bacterial number of 10^6^ cfu/mL on the 96-well plate and incubate it for 18 h at 35 ± 1 °C, as described before [47]. MIC was defined as the lowest concentration without visible growth.

#### 4.1.4. Hemolysis Assay

Hemolytic activity was determined by the hemoglobin release assay on a 96-well plate. Fresh venal blood was collected from volunteers in anticoagulant tubes. The blood samples were centrifuged for 3 min at 2500 rpm, and afterwards, the supernatant was discarded and PBS (pH 7.4) was added. The tubes were gently inverted, and the previous step was repeated two times. Substances were serial two-fold diluted in PBS (pH 7.4) on a 96-well V-bottom plate. Triton X-100 (1% in PBS) was used as a positive control (X_pos_). One column did not contain any substance and was used as a blank (X_blank_). In total, 50 µL of the purified blood samples was added to all columns. After 2 h incubation at 37 °C, the 96-well plate was centrifuged at 4000 rpm for 2 min. The supernatant was transferred to a new flat-bottom 96-well plate and absorption was measured at 554 nm. The percentage of hemolysis was calculated according to the following equation:

% hemolysis = 100 × (X_sample_ − X_blank_)/(X_pos_ − X_blank_). (1)

#### 4.1.5. Cytotoxicity Studies

For the validation of possible side effects provoked by the conjugates, the substance-specific cytotoxicity was determined by the WST-1 assay ((4-[3-(4-Iodophenyl)-2-(4-nitro-phenyl)-2*H*-5-tetrazolio]-1,3-benzene). For these tests, HuH7 cells were seeded in 96-well plates (25,000 cells/well) and cultured overnight in a humidified atmosphere at 37 °C and 5% CO_2_. After the removal of medium, the cells were exposed to the compounds (diluted in medium). As a viability control, cells were treated with medium only and additionally, further cells were lysed with TRITON^®^ X-100 (no viability). After 3 h of incubation, the medium was removed and replaced by WST-1 containing medium. After 2 h, the absorption was measured at 450 nm. The percentage of viable cells was determined in relation to untreated cultured cell controls (=100% viability).

#### 4.1.6. Radiolabeling

For ^125^I-radiolabeling, the peptide sequence of V_V_-R_3_C-C_12_ was extended with an additional D-tyrosine. Conjugates were labeled as described previously by Uhl et al. [41]. Briefly, the substance was dissolved in phosphate buffer (0.25 M) at a concentration of 1 mg/mL. The desired amount of radioactive ^125^I was added to 25 µL of the dissolved compound before starting the reaction by adding 10 µL of a 1 mM chloramine T solution, as described previously [48]. After 30 s, the reaction was stopped by adding 20 µL of a saturated methionine solution. Substances were subsequently purified by semi preparative RP-HPLC. For analysis, a radio-HPLC was conducted (Agilent 1100 series) using a Chromolith^®^ Performance RP-18e, 100 × 3 mm column, applying a linear gradient of 0.1% TFA in water (eluent A) to 0.1% TFA in acetonitrile (eluent B) within 5 min; the flow rate was 2 mL/min and the UV absorbance λ = 214 nm, with γ-detection.

#### 4.1.7. Molecular Imaging and Biodistribution Studies

All animal trials were approved by the Animal Care and Use committees at the University of Heidelberg, Heidelberg, Germany and the Regierungspräsidium Karlsruhe, Germany. Adult female Wistar rats (200–250 g) were purchased from Janvier Labs (Le Genest-Saint-Isle, France). For injection of the labeled compounds, the animals were anaesthetized by isoflurane inhalation and the radiolabeled substances, dissolved in 100 μL of 0.9% NaCl, were injected into the tail vein (1–3 megabecquerel (MBq) for molecular imaging and 0.5 MBq for biodistribution studies). Scintigraphic images were recorded at 10 min, 1 h, 2 h, 3 h, 5 h, 24 h, and 96 h post administration by a γ-camera.

For biodistribution studies, the rats were sacrificed at the determined time points. The respective organs were removed and weighted. The radiation emitted by each organ was measured by a Cobra Auto γ-Counter in comparison with corresponding standards. Considering the injected dose (ID), organ weight (in g), and γ-count, the percentage of activity was determined as % ID/g.

## 5. Conclusions

The increasing number of bacterial resistances poses many unprecedented problems to humanity. The development of novel, highly active antibiotics, especially against multidrug-resistant strains, is therefore more important than ever before. In this study, we combined recently published approaches of vancomycin modifications in order to investigate the structure–activity relationship of the synthesized conjugates with respect to the antimicrobial activity on vancomycin-resistant enterococci. The data supports the use of lauric acid in lipopeptide conjugates and highlights the importance of the modification site. Therefore, other structural modifications could be investigated with varying derivatization strategies.

## Supplementary Materials

The following are available online at https://www.mdpi.com/1424-8247/13/6/110/s1, Table S1: Tested conjugates, their respective MIC median values and MIC range against the vanA-resistant, clinical isolate of *E. faecium* UL602570 are shown; Table S2: The tested conjugates, their respective MIC median values and MIC range against the vanA-resistant strain of *E. faecium* ATCC 51559, the vanB-resistant strain of *E. faecalis* ATCC 51299 and the vanC-resistant strain of *E. casseliflavus* ATCC 700327 are shown.; Table S3: The conjugates evaluated in the hemoglobin release assay, applied concentrations and the respective hemoglobin release are shown.; Table S4: The tested conjugates, their respective viability and concentration (evaluated on HuH7 cells) are shown.
