# Supplementary material for: Vancomycin-Lipopeptide Conjugates with High Antimicrobial Activity on Vancomycin-Resistant Enterococci
Source: Pharmaceuticals (Basel). 2020 May 29;13(6):110. doi: 10.3390/ph13060110 (PMC7345083; doi:10.3390/ph13060110)
Supplement: Supplementary file 1 [file pharmaceuticals-13-00110-s001.pdf]

Supplementary Information:

"Vancomycin-Lipopeptide Conjugates with High Antimicrobial Activity on Vancomycin-Resistant Enterococci"

Table S1: Tested conjugates, their respective MIC median values and MIC range against the vanA-resistant, clinical isolate of *E. faecium* UL602570 are shown.

Compound	MIC median [mg/L]	MIC range [mg/L]
V_V-R_3C	4.35	4.35 – 8.69
V_V-R_3C-C_6	4.63	2.31 – 9.25
V_V-R_3C-C_8	1.49	0.30 – 2.39
V_V-R_3C-C_{10}	0.63	0.05 – 0.84
V_V-R_3C-C_{12}	0.24	0.06 – 0.48
V_V-R_3C-C_{14}	0.46	0.31 – 0.62
V_V-R_3C-C_{16}	2.50	2.50 – 3.50
V_V-R_3C-C_{18}	2.54	1.27 – 2.54
V_V-R_3yC-C_{12}	1.02	0.25 – 2.03
V_N-R_3C	67.75	32.00 – 67.75
V_N-R_3C-C_6	72.00	36.00 – 72.00
V_N-R_3C-C_8	18.31	9.16 – 18.31
V_N-R_3C-C_{10}	4.66	4.66 – 9.31
V_N-R_3C-C_{12}	2.37	2.37 – 4.73
V_N-R_3C-C_{14}	2.41	2.41 – 4.81
V_N-R_3C-C_{16}	2.45	2.45 – 4.89
V_N-R_3C-C_{18}	4.97	2.48 – 4.97
V_C-R_3C	34.75	17.38 – 34.75
V_C-R_3C-C_6	106.00	-
V_C-R_3C-C_8	9.54	9.54 – 19.08
V_C-R_3C-C_{10}	4.77	4.77 – 9.53
V_C-R_3C-C_{12}	4.84	2.42 – 4.84
V_C-R_3C-C_{14}	2.45	2.45 – 4.91
V_C-R_3C-C_{16}	5.00	5.00 – 10.00
V_C-R_3C-C_{18}	10.17	5.08 – 10.17

Table S2: The tested conjugates, their respective MIC median values and MIC range against the vanA-resistant strain of *E. faecium* ATCC 51559, the vanB-resistant strain of *E. faecalis* ATCC 51299 and the vanC-resistant strain of *E. casseliflavus* ATCC 700327 are shown.

Compound	ATCC 51559		ATCC 51299		ATCC 700327	
	MIC median [mg/L]	MIC range [mg/L]	MIC median [mg/L]	MIC range [mg/L]	MIC median [mg/L]	MIC range [mg/L]
V_V-R_3C-C_{12}	3.88	3.88 – 7.75	4	-	2.5	0.5 – 4
V_N-R_3C-C_{12}	4.72	4.72 – 9.44	4	-	2.5	1 – 4
V_C-R_3C-C_{12}	4.84	4.84 – 9.69	4	-	4	1 – 4

Table S3: The conjugates evaluated in the hemoglobin release assay, applied concentrations and the respective hemoglobin release are shown.

Compound	Concentration [µM]									
	**833.00**	**416.50**	**208.25**	**104.13**	**52.06**	**26.03**	**13.02**	**6.51**	**3.25**	**1.63**
	**Hemoglobin release [%]**									
V_{V}-R_{3}C-C_{6}	4.77 ± 1.92	1.38 ± 0.53	0.55 ± 0.59	0.08 ± 0.85	-0.08 ± 0.66	0.18 ± 0.40	-0.06 ± 0.36	-0.29 ±0.50	-0.13 ± 0.48	-0.41 ± 0.53
V_{V}-R_{3}C-C_{12}	6.01 ± 1.54	4.02 ± 0.58	0.93 ± 1.04	0.64 ± 0.68	0.53 ± 0.63	0.43 ± 0.71	0.06 ± 0.56	0.08 ± 0.64	-0.02 ± 0.58	0.31 ± 0.94
V_{V}-R_{3}C	15.32 ± 2.21	2.13 ± 0.52	0.14 ± 0.83	-0.11 ± 0.41	-0.15 ± 0.49	0.08 ± 0.66	-0.42 ± 0.44	-0.29 ± 0.44	-0.14 ± 0.47	0.24 ± 0.42
V_{N}-R_{3}C-C_{12}	11.68 ± 5.56	4.02 ± 1.76	1.78 ± 0.37	2.07 ± 1.00	0.58 ± 0.39	0.32 ± 0.33	0.11 ± 0.29	0.24 ± 0.24	-0.01 ± 0.18	-0.05 ± 0.29
V_{C}-R_{3}C-C_{12}	21.83 ± 2.60	8.45 ± 4.58	4.70 ± 1.60	2.38 ± 1.40	0.78 ± 0.48	0.61 ± 0.35	0.34 ± 0.22	0.46 ± 0.28	0.10 ± 0.16	0.16 ± 0.59
Vancomycin	0.55 ± 0.32	0.34 ± 0.48	0.38 ± 0.43	0.19 ± 0.28	0.19 ± 0.23	0.28 ± 0.33	0.08 ± 0.26	-0.01 ± 0.23	0.00 ± 0.19	-0.04 ± 0.19
**Compound**	**Concentration [µM]**									
	**799.00**	**399.50**	**199.75**	**99.88**	**49.94**	**24.97**	**12.48**	**6.24**	**3.12**	**1.56**
	**Hemoglobin release [%]**									
V_{V}-R_{3}C-C_{18}	14.82 ± 2.04	11.62 ± 1.58	8.26 ± 1.17	4.61 ± 1.10	1.03 ± 1.18	-0.24 ± 0.40	-0.28 ± 0.36	-0.22 ± 0.37	-0.45 ± 0.40	-0.21 ± 0.34

Table S4: The tested conjugates, their respective viability and concentration (evaluated on HuH7 cells) are shown.

Compound	Concentration							
	**8x MIC**		**4x MIC**		**2x MIC**		**1x MIC**	
	**Conc. [mg/L]**	**Viability [%]**	**Conc. [mg/L]**	**Viability [%]**	**Conc. [mg/L]**	**Viability [%]**	**Conc. [mg/L]**	**Viability [%]**
V_{V}-R_{3}C-C_{6}	37.04	108.25 ± 4.55	18.52	108.18 ± 4.33	9.26	108.10 ± 3.73	4.63	110.27 ± 1.30
V_{V}-R_{3}C-C_{12}	1.92	99.37 ± 0.80	0.96	101.72 ± 6.94	0.48	100.74 ± 4.90	0.24	107.55 ± 0.96
V_{V}-R_{3}C-C_{18}	20.00	91.97 ± 3.06	10.00	100.40 ± 2.43	5.00	103.72 ± 5.62	2.50	104.43 ± 1.87
V_{V}-R_{3}C	34.80	98.33 ± 0.71	17.40	105.10 ± 3.48	8.70	108.49 ± 6.90	4.35	113.03 ± 6.02
